# An Atypical Case of Intracranial Dermoid Cyst in an Adult Female: A Case Report and Literature Review

**DOI:** 10.7759/cureus.39807

**Published:** 2023-05-31

**Authors:** Sanni Emmanuel, Pugazhendi Inban, Ogbonnaya Akuma, Muhammad Nouman Aslam, Fawad Talat, Ammarah Nizamani, Venkata Sai Harshabhargav Chenna, Ebene Mbende Romain, Carlo Kristian Chu Carredo, Aadil Khan

**Affiliations:** 1 Surgery, National Hospital Abuja, Abuja, NGA; 2 General Medicine, Government Medical College, Omandurar Government Estate, Chennai, IND; 3 Internal Medicine, Ebonyi State University, Abakaliki, NGA; 4 Internal Medicine/Sleep Medicine, Midwest Sleep and Wellness Clinic, Chicago, USA; 5 Internal Medicine, King Edward Medical University, Lahore, PAK; 6 Internal Medicine, Liaquat University of Medical and Health Sciences, Jamshoro, PAK; 7 Medicine, University of Perpetual Help System DALTA (Daisy Antonio Laperal Tamayo), Las Piñas, PHL; 8 Internal Medicine, Faculty of Medicine and Pharmaceutical Sciences, University of Douala, Douala, CMR; 9 Internal Medicine, Cebu Institute of Medicine, Cebu, PHL; 10 Internal Medicine, Lala Lajpat Rai Hospital, Kanpur, IND

**Keywords:** congenital dermoid cysts (cds), chemical meningitis, ectodermal, seizures, intracranial dermoid cysts

## Abstract

Intracranial dermoid cysts are unusual cystic tumors that are often benign, develop slowly, and are present from birth. They are made up of mature squamous epithelium and may house ectodermal features such as glands (apocrine, eccrine, and sebaceous). Dermoid cysts may be asymptomatic and can be detected incidentally during brain imaging for unrelated causes. Dermoid cysts tend to grow gradually and may eventually exert pressure on the brain and surrounding areas. Unfortunately, they can seldom burst, resulting in an unfavorable prognosis for the patient depending on the size, location, and clinical presentation. Headache, convulsions, cerebral ischemia, and aseptic meningitis are the most frequent symptoms. Magnetic resonance imaging (MRI) and computed tomography (CT) of the brain aid in accurate diagnosis and therapy planning. In some cases, the treatment consists of surgical monitoring with regular surveillance imaging. In other cases, surgery is needed, depending on the symptoms and the location of the cyst in the brain.

## Introduction

Intracranial dermoid cysts are rare tumors that develop from ectopic epithelial cells. The entrapment of the surface ectoderm along the lines of embryonic fusion gives rise to congenital dermoid cysts (CDS) and can keep expanding. With this ability for continual development, proper monitoring of its extension to epidural space and a plan for removal should be highly considered [1]. Dermoid cysts contain lipid material that makes up the dermoid cyst with fluid centrally and tissue peripherally arranged. They are benign, slow growing, and rarely rupture. However, it can lead to complications due to compression of the surrounding region or vasculature. In rare cases, spontaneous rupture of these cysts can lead to chemical meningitis due to fat droplet dissemination. It can also lead to new-onset seizures [2]. Despite not being a rapidly growing tumor and considered benign, dermoid cysts show some signs and symptoms which are neurological by impacting the surrounding nerves and blood vessels and do not readily rupture [3]. The intracranial dermoid cyst appears as hypodense cystic lesions on computed tomography (CT) imaging and hyperintense on magnetic resonance imaging (MRI) due to the fatty component [4].

## Case presentation

A 54-year-old female was brought to the emergency department after two episodes of abnormal tonic-clonic seizures, followed by altered sensorium, and frothing from the mouth and incontinence. It was her third episode in the past two days. No significant past medical history was present. No contributory family history was present, and she had no history of substance or alcohol abuse.

On physical examination, the patient was afebrile, not oriented to time, place, and person, with a heart rate of 65/min, blood pressure of 140/70 mmHg, and respiratory rate of 15/min. Her systemic examination, including neurological examination, was unremarkable except for a headache. She had no signs of meningeal irritation or involvement of cranial nerves. Initial laboratory investigations were normal. A provisional diagnosis of seizure disorder was made, as evidenced clinically. The patient was started on levetiracetam 500 mg twice times a day and clobazam 10 mg twice a day for new-onset seizures. Electroencephalography (EEG) was unremarkable with most of the waves of 8hz and higher frequencies.

**Figure 1:**
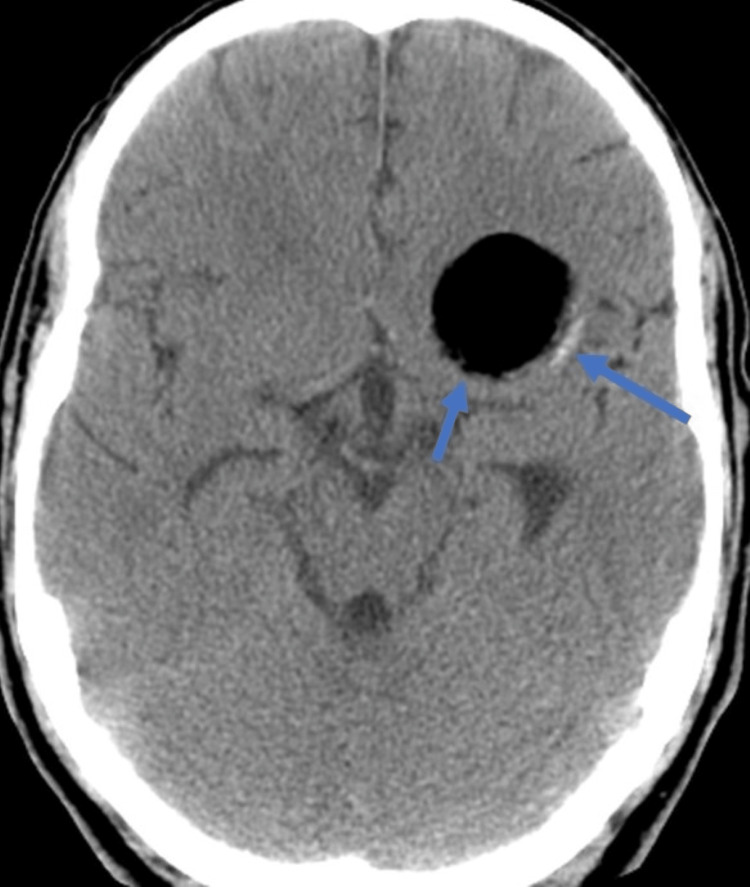
Brain CT revealing a hypodense lesion with smooth margins having cystic component and linear calcifications. CT: computed tomography.

**Figure 2:**
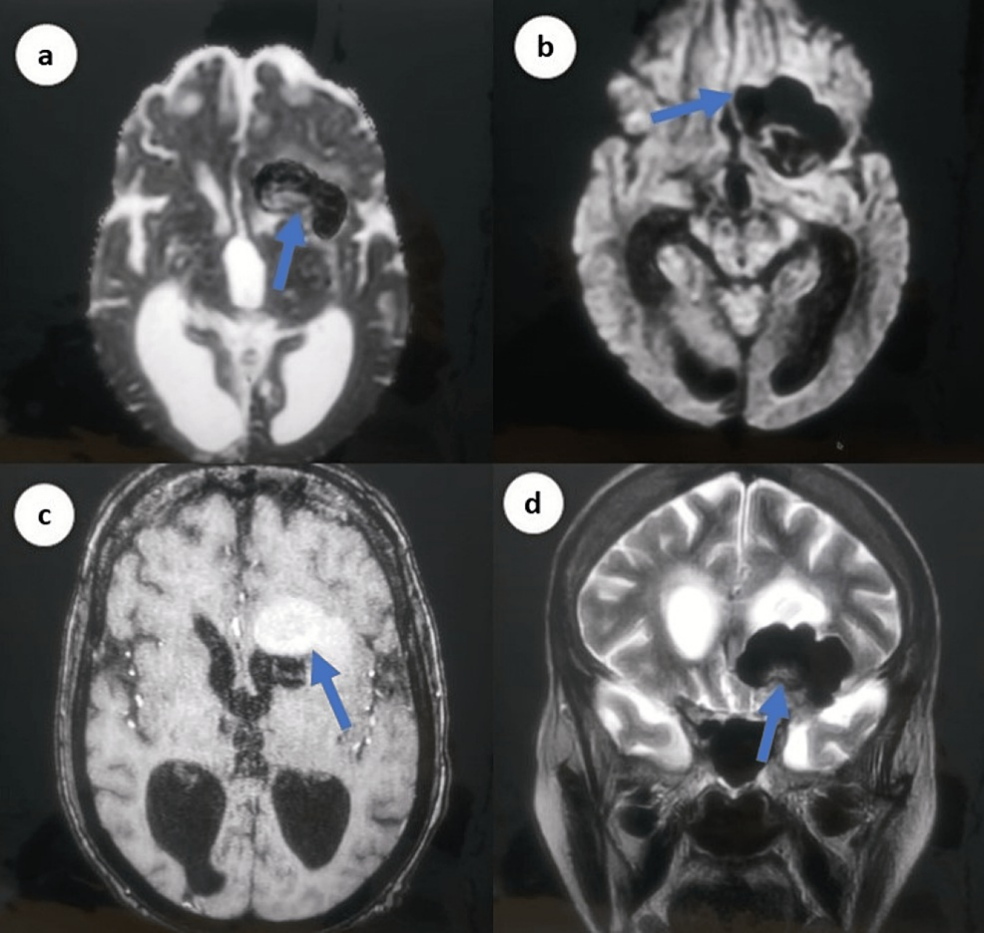
Brain MRI demonstrating a high-intensity cystic lesion in the middle and anterior cranial fossa with thin marginal enhancement and calcifications and area of gliosis was visualized in the right capsular, periventricular regions, and right occipital region with ex vacuo dilatation of the occipital horn of the right lateral ventricle: (a) FLAIR, (b) DWI), (c) T1, (d) T2 MRI: magnetic resonance imaging, DWI: diffusion-weighted imaging, FLAIR: fluid attenuated inversion recovery

CT scan of the brain revealed a hypodense lesion with a cystic component, linear calcifications, and smooth margins (Figure 1). In addition, using MRI, the fluid-attenuated inversion recovery (FLAIR) sequence T1 and T2 and restricted diffusion on the diffusion-weighted imaging (DWI) sequence revealed a hyperintense cystic lesion with thin marginal enhancement in anterior and middle cranial fossa with midline shift. In addition, it showed an area of gliosis in the right capsular, periventricular, and occipital region with ex vacuo dilation of the occipital horn of the right lateral ventricle, suggestive of chronic infarcts. There was diffuse cortical atrophy (Figure 2).

After integration, clinical and radiological features do not fit aneurysm or meningioma. However, after further consultation from the neurosurgery department, the diagnosis of the intracranial dermoid cyst was elucidated based on distinguishing features on CT and MRI images. Informed surgical consent was taken from the patient for the neurosurgical procedure and she was taken for craniotomy for surgical removal of the cystic lesions. The cystic components and their capsule were removed successfully and sent for histopathology, which confirmed dermoid cysts with stratified squamous epithelium associated with adipose tissue, keratinous debris, calcific depositions, and cholesterol pockets. Postoperatively, the patient remained neurologically intact. An MRI scan of the brain obtained three months after resection showed minimal persistent intraventricular fat droplets without recurrence or discernible complications of the resection. The patient has resumed most normal activities.

## Discussion

Dermoid cysts seldom cause symptoms. The primary presenting complaints are seizures and headaches, which are connected to its mass-effect features [4]. Despite being benign, slow-growing tumors that do not readily rupture, these tumors can show signs depending on the location and compression of surrounding regions and blood vessels [3]. In this case, a patient with an intracranial dermoid cyst presented with new-onset seizures. However, she had no history of seizures.

Dermoid cysts arise from ectopic epithelial cells [5]. These are rare tumors and constitute 0.7%. of all intracranial tumors and are derived from ectodermal tissue earlier than the sixth week of embryogenesis into the neural tube through cell inclusion [6]. This is why the midline is their common location and is commonly found in the frontonasal, sellar, parasellar, and posterior fossa regions [7]. Dermoid cyst is very uncommon in the temporal lobe. Males are slightly more affected than females [6,7]. Dermoid cysts occur in different age groups and have been noted to be more predominant in children below 10 years and adults between the fourth and sixth decade of life [8]. Our patient was a 54-year-old female and was consistent with the predominant age. However, this case was atypical with the presence of a dermoid cyst in the middle and anterior cranial fossa involving the frontal, parietal, and temporal lobes rather than in the midline.

Radiologically, intracranial dermoid cysts can be easily noticed. The hypodense appearance of dermoid cysts on CT is due to their lipid content [9]. Dermoid cysts can show up as hyperdense tumors on both T1-weighted and FLAIR sequences of an MRI. On T2-weighted sequences, they can show up as either hypodense or inhomogeneously hyperdense. Our patient’s CT scan showed a large lesion with calcification in the left temporal region. This finding prompted an MRI brain, which revealed a small lesion showing a hyperintense signal on the FLAIR sequence. The imaging is in keeping with the atypical dermoid cyst pattern based on the location and size of the cyst [10].

Dermoid cysts, when symptomatic, can be surgically excised. Recurrence is uncommon, provided that complete excision is achieved. Sometimes, due to the local adhesion of the capsule to vital structures, incomplete excision must be performed [11]. There is the possibility of spontaneous rupture of dermoid cysts that presents without symptoms, and when this happens, the patient is at risk of chemical meningitis and hydrocephalus, which can be an indication for surgical intervention in large tumors [12]. For supratentorial region dermoid cyst, rich in lipid content, there is a likelihood of rupture; therefore, surgical treatment or close observation is to be considered. Surgical steps of resection of dermoid cysts are incision of the capsule, internal debulking with removal of cyst contents, and microsurgical dissection of the capsule from adherent or adjacent neurovascular structures. In many cases, the dermoid capsules have a dense adherence to the brain parenchyma and vasculature and so a total removal is very difficult [13]. Extensive irrigation of the resection bed and subarachnoid cisterns is recommended to minimize the risk of postoperative aseptic meningitis and delayed cerebral ischemic deficit [12,13].

A total resection is a reasonable consideration if the location is favorable, to prevent seizures, and to send the sample for histopathological diagnosis. However, in cases where the tumor is located at either the suprasellar region or cerebellopontine angle, there may be a possibility of severe adhesion occurring between the tumor and the cranial nerves, brain stem, and vessels, and this will make complete resection of the tumor difficult, and in such situations, excessive resection should not be an option [7,8]. To prevent postoperative chemical meningitis, excessive caution should be exercised during cyst manipulation intraoperatively to avoid spreading contents of the cyst [9]. Clinical and radiological diagnosis is essential for accurate diagnosis of intracranial dermoid cysts, and surgical resection is the treatment of choice.

## Conclusions

Intracranial dermoid cysts are rare, benign, slow-growing cystic lesions but occasionally can cause complications depending on their size and location. Our case report accentuates a rare incidence, which presented emergently with seizures. Clinicians should be aware of the radiological pattern of intracranial dermoid cysts, hypodense on CT and hyperintense on MRI, which along with the location (usually midline) can aid in its diagnosis. Seizures, especially if they are not controlled, are a good indication of the removal of a tumor.
